# Supplementary figures and images for: Tip Growth Defective1 interacts with the cellulose synthase complex to regulate cellulose synthesis in Arabidopsis thaliana
Source: PLoS One. 2024 Feb 15;19(2):e0292149. doi: 10.1371/journal.pone.0292149 (PMC10868759; doi:10.1371/journal.pone.0292149)

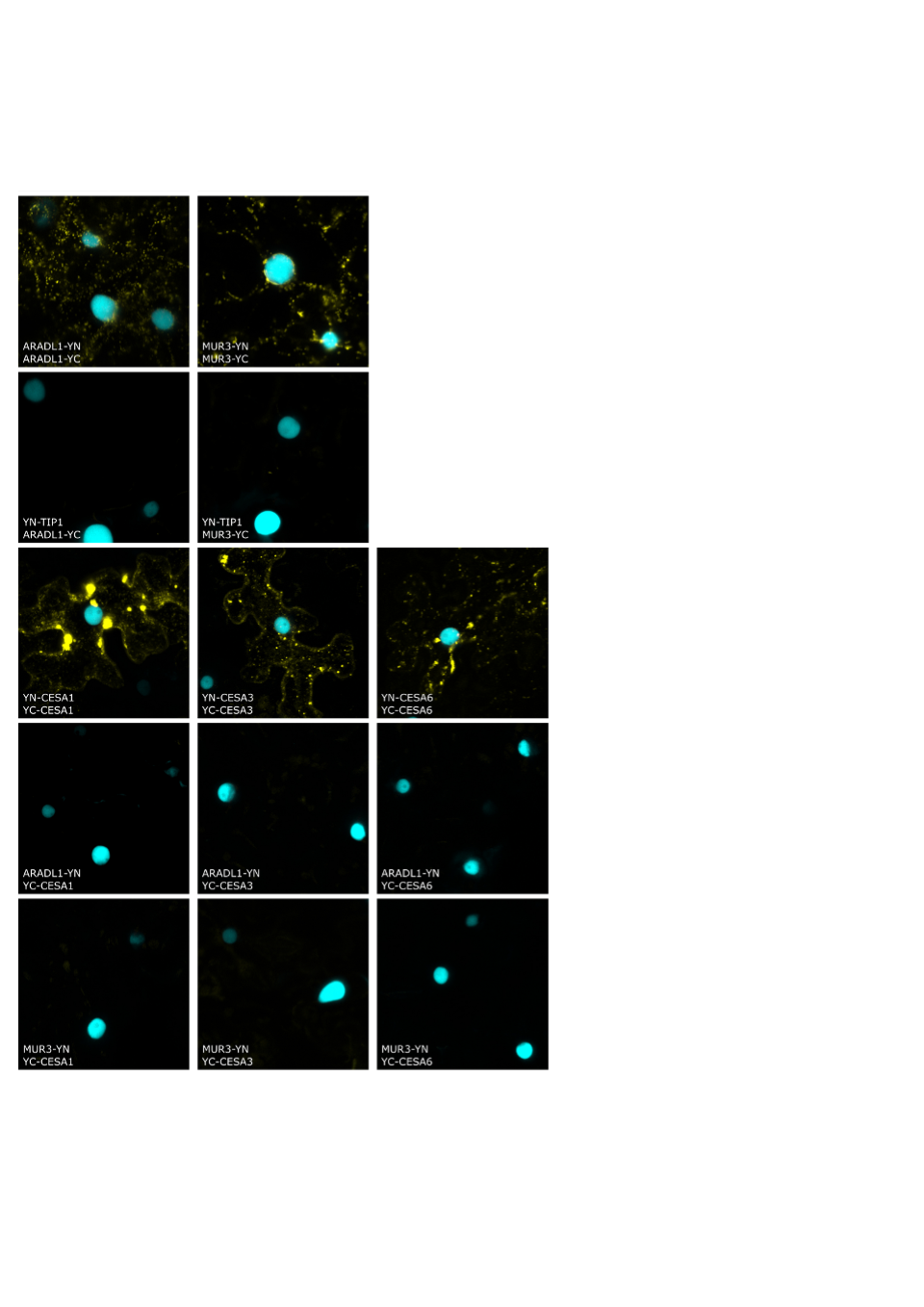

Supplement: S1 Fig — N-terminal YN or YC fusions of ARADL1 and MUR3 were used as controls and interacted only with themselves but not with any of the N-terminal YN or YC fusions of TIP1 or the CESAs (CESA1, 3 and 6). CESA1, 3 and 6 can dimerize as evidenced by fluorescence signal when co-expressing YN or YC fusions of CESAc with YN or YC fusions of the secondary wall CESAs The nuclear marker CFP-N7 (cyan) was used as a positive transformation control in all experiments. (TIF) [file pone.0292149.s001.tif]
